# Improvement of SCD morbimortality in children: experience in a remote area of an African country

**DOI:** 10.1186/s12913-021-06286-7

**Published:** 2021-04-01

**Authors:** Benoît Mukinayi Mbiya, Didier Kalenda Kalombo, Yannick Nkesu Mukendi, Valery Daubie, John Kalenda Mpoyi, Parola Mukendi Biboyi, Ghislain Tumba Disashi, Béatrice Gulbis

**Affiliations:** 1 Pediatrics Department, Faculty of Medicine, University of Mbujimayi, 06201 Mbujimayi, Democratic Republic of Congo; 2 Sickle Cell Reference Center, Clinique Pédiatrique de Mbujimayi, Pediatrics Clinic of Mbujimayi, 06201 Mbujimayi, Democratic Republic of Congo; 3 Clinical Biology Department, LHUB-ULB, Université Libre de Bruxelles, 1070 Brussels, Belgium; 4 Internal Medicine Department, Faculty of Medicine, University of Mbujimayi, 06201 Mbujimayi, Democratic Republic of Congo; 5 Clinical Chemistry Department, Hereditary Red Blood Cell Disorders, LHUB-ULB, Université Libre de Bruxelles, 1070 Brussels, Belgium

**Keywords:** Sickle cell disease, Diagnosis, treatment, follow-up, Democratic Republic of Congo

## Abstract

**Background:**

Sickle cell disease (SCD) is a public health problem in the Democratic Republic of Congo. While reference sickle cell centers have been implemented in capital cities of African countries and have proven to be beneficial for SCD patients. In the Democratic Republic of Congo, they have never been set up in remote areas for families with low or very low sources of income.

**Method:**

A cohort of 143 children with SCD aged 10 years old (IQR (interquartile range): 6–15 years) (sex ratio male/female = 1.3) were clinically followed for 12 months without any specific intervention aside from the management of acute events, and then for 12 months with a monthly medical visit, biological follow-up, and chemoprophylaxis (folic acid/penicillin), adequate fluids and malaria prevention.

**Results:**

The median age of patients at the diagnosis of SCD was 2 years (IQR: 1–5). The implementation of standardized and regular follow-ups in a new sickle cell reference center in a remote city showed an increase in the annual mean hemoglobin level from 50 to 70 g/L (*p* = 0.001), and a decrease in the lymphocyte count and spleen size (*p* < 0.001). A significant decrease (*p* < 0.001) in the average annual number of hospitalizations and episodes of vaso-occlusive crises, blood transfusions, infections, and acute chest syndromes were also observed.

**Conclusions:**

The creation of a sickle cell reference center and the regular follow-up of children with sickle cell disease are possible and applicable in the context of a remote city of an African country and represent simple and accessible measures that can reduce the morbimortality of children with sickle cell disease.

## Background

The world’s leading genetic disease, sickle cell disease (SCD) was declared a public health priority by the United Nations in 2008 during the 63rd session of the UN General Assembly. According to the study conducted by Piel et al. in 2017, Africa had more than 250,000 births of children with a severe form of this disease [1]. In the absence of early and appropriate care, 50 to 90% of these newborns will die before the age of five [2]. In Central Africa, the incidence of SCD at birth is estimated to be 1 to 2%—the highest in the world. In sub-Saharan Africa, after Nigeria, the highest number of births of children with SCD is the Democratic Republic of Congo (DRC) [3].

Of the estimated general population of 91,994,000, the DRC has 25–30% heterozygous for hemoglobin S, 2 to 3% of SCD newborns which account for an annual number estimated to 40,000 [4, 5]. Without major changes in the expansion of this endemic disease, the number of subjects with SCD is growing at an uncontrolled rate [6, 7]. While these figures are significant from an epidemiological point of view, the disease remains poorly recognized and can be neglected or lead to underdiagnosis resulting in high mortality and morbidity [6, 8]. Despite significant progress in reducing the under-five mortality rate in the DRC between 2014 and 2018 [9, 10], SCD is still characterized by a very high rate of morbidity and mortality [2, 11]. Treatment with hydroxyurea is feasible and safe for SCD children living in sub-Saharan Africa and not only reduces mortality but also reduces various complications like vaso-occlusive events [12]. However, without government intervention or another source of funding, its cost is prohibitive in most of the African regions [13].

SCD is a major public health concern in the DRC, and the basic resources for its management have remained insufficient with most of the initiatives that have been conducted only in Kinshasa.

The application of simple, accessible, and less expensive measures accompanied by regular medical follow-up would improve the health of patients with SCD in sub-Saharan Africa if it were applied rigorously [14]. In high-income countries, the increased survival and better quality of life of patients with SCD are less due to sophisticated therapies, such as stem cell transplantation, but more to organized support systems with early and adequate patient care [15, 16]. A preliminary survey carried out in 2015 on the knowledge and behavior of 50 families affected by SCD in Mbujimayi showed that the children had a severe form of the disease, that there was no reference center, and that families had little knowledge of the disease. The majority (96%) of families wanted the creation of a reference center and 94% of them had agreed to subscribe to it for the medical follow-up of their sick children. In order to improve the care of sickle cell patients in Mbujimayi, the creation of a reference center and the possibility of a fixed annual amount for this care were the objectives set [17].

The objective of this study is to demonstrate the feasibility and accessibility of adapted care for children with SCD from low-income families which are based on the creation of a reference center in a remote town in the DRC.

## Methods

### Study context

**Fig. 1:**
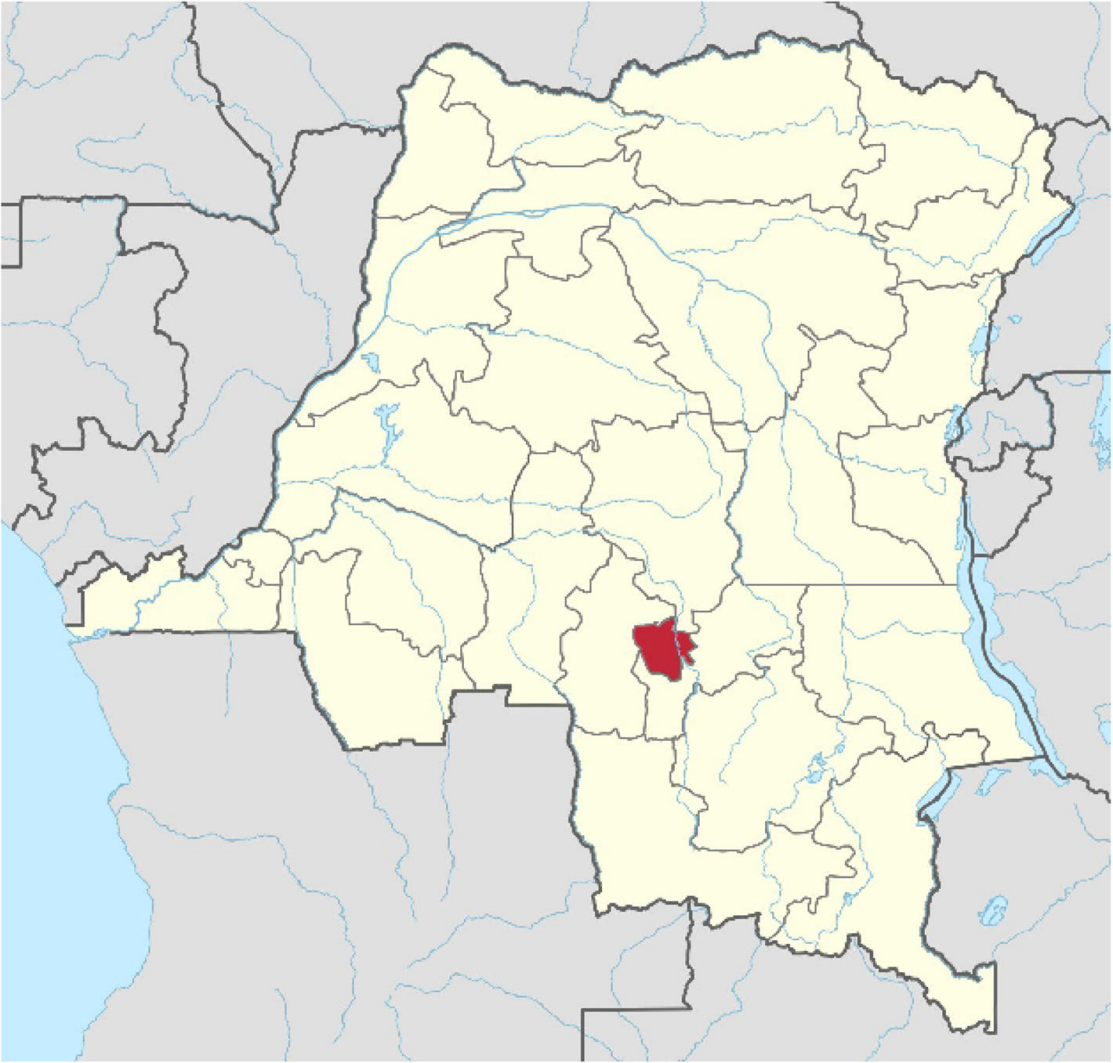
Location of the city of Mbujimayi on the map of the Democratic Republic of Congo [8]

This study was conducted in Mbujimayi, which is the capital city of the province of Eastern Kasai in the DRC (Fig. 1) [18]. It is the third-largest city in terms of population. Mbujimayi’s 2020 population is now estimated to be 2,525,263 (Mbujimayi population data 2020), covering an area of 135.12 km^2^ and corresponding to a population density of 12,441 inhabitants/km^2^.

In the DRC, SCD management is devoted to the PNLCD. In line with the strategy for strengthening the health system, the PNLCD recommendations have not integrated SCD control activities in primary healthcare structures. The PNLCD is also not present in Mbujimayi.

The study was carried out in the Mbujimayi Pediatric Clinic—one of the largest specialized pediatric structures. This clinic represents a vertical program which is a private philanthropic program not integrated into the primary health care of the health system in the DRC. Currently, this clinic organizes the management of SCD with regular follow-ups at no cost to patients. It includes a pediatric ward with a capacity of 20 beds that are constantly occupied, a neonatology ward, maternity ward, and semi-automated laboratory. However, it is important to note that the city of Mbujimayi has also two large old hospitals (Bonzola General Reference Hospital and Dipumba General Reference Hospital). Both hospitals are owned by the Société Minière de Bakwanga (MIBA), the first industrial diamond production company in the DRC. Due to the fall of MIBA more than 15 years ago, the company is facing very serious financial difficulties. These hospitals have been abandoned and are in an advanced state of disrepair, requiring rehabilitation. This has a significant impact on the quality of care to patients, including sickle cell patients. In the case of acute complications, sickle cell patients are treated at their own expense and there is generally no follow-up. With the opening of the Mbujimayi pediatric clinic and the organization of free care, the influx of sickle cell patients has increased significantly.

### Study type and inclusion criteria

From January 2017 to December 2018, we conducted an open-label clinical trial (registered as such in the ethics committee document) including SCD children. These children had never been followed up or treated with hydroxyurea before their inclusion in the study. The cohort was followed up for 2 years, consisting of a simple follow-up in the first year (2017) without the application of recommendations for the management of SCD, and in the second year (2018), with a classic regular follow-up of the same cohort with the application of simple, classic, and locally accessible recommendations (Table 2).

Patients diagnosed with SCD (i.e., sickle cell anemia or sickle cell/beta-thalassemia) who were older than 3 months and younger than 18 years were eligible for this study. Children under 3 months of age were excluded because of the lack of routinely organized neonatal screening to identify newborns with pre-symptoms. The diagnosis of SCD was made using an isoelectrofocusing (IEF) technique (Perkin Elmer, Massachusetts, USA). Blood samples were collected on blotting paper and taken from the laboratory of the Monkole Hospital in Kinshasa, located at the cape of the DRC. Monkole Hospital is a large center of SCD in Kinshasa, the DRC. In addition, patients considered for inclusion had to reside in Mbujimayi during the study period. We excluded all SCD children whose primary guardian refused to sign to indicate informed consent as well as those who were heterozygous AS.

### Data collection

Data were collected during follow-up consultations using a data collection sheet for each patient. Then, these data were transcribed into a common database (Excel file).

### Study parameters and operational definitions

Comprehensive health care that included monthly medical visits, usual care for a sickle cell patient such as crisis prevention (antibioprophylaxis, folic acid, deworming and anti-malarial prophylaxis and counseling, etc.), regular biological and clinical follow-up.

Sociodemographic parameters were age, sex, and age at the first diagnosis of SCD (recorded by a healthcare provider).

Clinical parameters were the origin of diagnosis (clinical and/or biological), weight (kg), height (cm), weight-for-height Z-score (WHO) assigned by sex and age, symptoms, and palpable size of the spleen. The clinical classification of splenomegaly according to Hackett’s grade includes five categories ranging from 0 to 5: from non-palpable spleen, even in deep inspiration (category 0; H0) to spleen descending well below the navel, exceeding the line passing between the umbilicus and the pubic symphysis (category 5; H5). Study Parameters and Operational Definitions (Table 1).

**Table 1:** Study Parameters and Operational Definitions

Parameters	Operational Definitions
Anemia	The decrease in whole-blood hemoglobin concentration of more than 2 standard deviations below the mean of age- and sex-matched reference range [19].
VOC	Any painful episode requiring the intake of an analgesic (e.g., paracetamol, ibuprofen, or tramadol) or leading to a medical consultation in a healthcare structure.
Infectious episode	Any noted increase in the body temperature beyond 38.5 °C needed to be managed in a healthcare facility.
Red blood cell transfusion	Any administration of labile blood products (in particular, packed red blood cells or whole blood) that occurred in a healthcare facility.
Acute chest syndrome	Presence of fever, cough, chest pain, difficulty breathing ± performance of chest X-ray.
Jaundice	A clinical observation, i.e., the presence of yellow coloration of the bulbar conjunctiva.
Hospitalization	Admission to hospital for treatment lasting at least 24 h.
Adherence to care	Assessed as excellent, fair, or poor depending on the clinical follow-up as observed.
Large city	A city with an urban landscape and an international airport that is directly connected to foreign countries.
Remote city	Urban-rural town in the country with no direct contact with foreign countries.

*VOC* vaso-occlusive crisis

Biological parameters were obtained by testing patients using an IEF technique and a hemogram (excluding information related to reticulocyte counts). The biological parameters, especially hemoglobin, were used to evaluate the severity of the disease.

Therapeutic parameters consisted of medications taken. The criteria for prescribing hydroxyurea comprised three or more severe vaso-occlusive crises occurring in the last 12 months, SCD-related pain, or chronic anemia interfering with daily activities, and severe or recurrent episodes of acute chest syndrome [20, 21].

### Medical monitoring

Patients were subjected to a 2-year follow-up process comprising a monthly planned medical visit with a clinical and hematological assessment. We opted for monthly visits given the local socio-cultural context. People are not used to seeing doctors outside of an acute medical situation. This strategy has enabled us to ensure that we are in constant contact and have a close assessment of patients and to increase awareness of the importance of medical monitoring, disease, and crisis prevention measures.

During the first year (2017), medical staff (doctors, nurses) and community relays received training on diagnosis, management of SCD, and the implementation of standardized follow-up in year 2. The first year passed without the application of any recommendations, while for the second year (2018), the systematic application of standardized and regular follow-ups was organized (Table 2).

**Table 2:** Follow-up of sickle cell patients and the applied strategy

Parameters of medical monitoring	Year 1	Year 2
Parental Counseling and Education [20, 22–26]	• Early identification of fever, VOC, anemia, broad spleen, and urgent consultation for management. • Report any acute events (VOC, acute anemia, fever, etc.).	• Education on the need for adequate nutrition, hydration, and regular hospital follow-ups. • Early identification of fever and its urgent treatment, and of a large spleen. • Use of prophylactic medications such as penicillin V, antimalarial drugs (sulfadoxine-pyrimethamine every 2 weeks, and deworming with mebendazole (once every 6 months).
Immunization [27]	• Checking the vaccine schedule. • No stimulation to get full vaccination coverage.	Immunization against infections according to the vaccines recommended in the DRC*: • Bacillus Calmette–Guérin vaccines against tuberculosis. • Diphtheria, tetanus, and pertussis. • Oral polio. • Measles. • Yellow fever. • Tetanus. • *Haemophilus influenza* type b; Pneumococcus (Prevenar 13).
Strategy	• Setting up a notification book of acute complications (fever, pain, acute anemia, etc.) that contains the contact number. • Organize free-of-charge emergency treatment for all SCD patients.	• Establishment of an appointment book to be given to parents or the patient: this book contained the dates of the appointments and the contact numbers. • Organize free-of-charge consultations for all SCD patients. • Set up a system of SMS and/or phone calls to remind people about appointments. • Organization of listening and information sessions for parents and patients every 3 months. • Monthly distribution of folic acid and oral penicillin. Mebendazole and antimalarial treatments were dependent on the patient.

Year 1: Follow-up of acute complications and biological parameters without application of conventional recommendations. Emergency support only. Year 2: Standardized and regular follow-up with the implementation of recommendations for the management of SCD [24] adapted to local conditions

*VOC* vaso-occlusive crisis, *SCD* SCD, *SMS* short message service. NB: No patients were treated with hydroxyurea because of the drug’s low availability and high cost

* The minimum vaccine coverage proposed/funded in the DRC [27]

### Statistical analysis

To analyze blood parameters of patients from children through to adulthood, data were reported as the percentage of the lower normal limit for corresponding age. Because no criteria are defined for our area in Congo, normal values were compiled from neighboring regions [28–35]. Normal distribution was tested with the D’Agostino and Pearson normality test. A paired t-test (Gaussian data) or Wilcoxon matched-pairs signed-rank test (no Gaussian data) were used to compare data for each patient. Data were plotted within boxplots (2.5–97.5% range). Spleen size evolution was analyzed with the Chi-square test and the relation between platelet count and spleen size was analyzed by dividing platelet counts by a re-encoded spleen size (h0 = 1, h1 = 2, …). *p* < 0.05 was considered to indicate statistical significance (* *p* < 0.05, ** *p* < 0.01, *** *p* < 0.001). Statistical analyses were performed using Prism 8.0.1. (GraphPad Software Inc., San Diego, CA) software.

### Ethical approval

All parents or legal respondents of patients provided their written informed consent for participation in the study. The study protocol had been reviewed and approved by the Ethics Committee of the Medical Faculty at the University of Mbujimayi (N/Réf.: 012/VD-RSCU/Fac-Méd/UM/DMT/2019) and the Head Board of the Public Health Division of Eastern Kasai Province (DPSPN°71/204/C.N.E. S/DPS/NTK/K.OR/2019). The study was conducted in agreement with the principles of the Helsinki Declaration II. The aim and procedures of the study were explained to the participants and legal respondents. The participants were informed that they could withdraw at any time without further obligation. The anonymity of the participants was guaranteed, and no personal details were recorded. The results of this study were presented to parents and legal respondents during a discussion session at the end of the study.

## Results

**Fig. 2:**
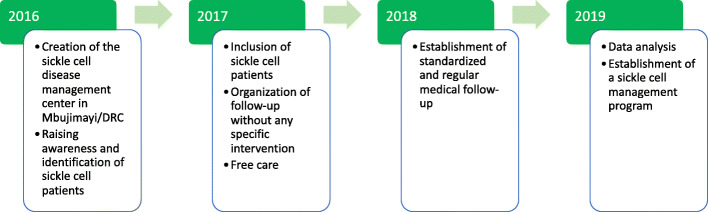
The overall evolution of the study on the application of standardized and regular follow-ups at Mbujimayi in the DRC

**Fig. 3:**
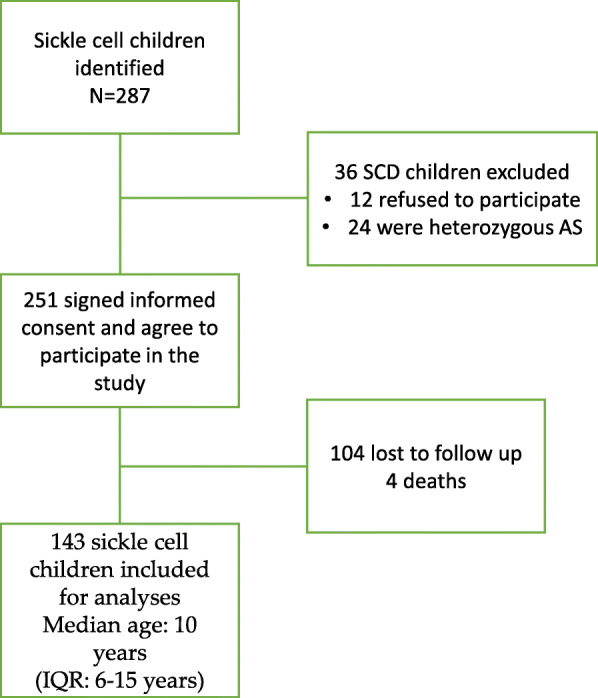
Inclusion of sickle cell children in the study

The overall evolution of the study on the application of standardized and regular follow-ups is described in Fig. 2 and the process of the recruited cohort of 143 SCD children is described in Fig. 3. The losses during follow-ups and the death rates during the first-year period were 41% (104/251) and 1.6% (4/251), respectively.

The demographic and clinical characteristics of SCD children at 12 months of follow-up (FU) before the implementation of international recommendations are reported in Table 3. The diagnostic means initially used were predominantly based on clinical features (43%, 62/143). The circumstances of the diagnosis were mostly VOC (66%) and anemia (18%), and none were diagnosed during the neonatal period. The Z-score weight for height less than −2DS was observed in 47% of the patients.

**Table 3:** Demographic and clinical characteristics of sickle cell children at 12 months of follow-up, before the implementation of sickle cell management recommendations (*n* = 143)

Variable	Median (IQR 25–75%)	n	%
Age	10 (IQR: 6–15 years)		
Age at diagnosis	2 (IQR: 1–5 years)		
	Before 1 year	53	37
	Between 1 and 5 years old	55	39
	Between 5 and 10 years old	22	15
	After 10 years	13	9
Sex	Female	62	43
Schooling	Yes	99	69
Z-score weight-for-height less than −2SD	Yes	67	47
Mode of the first diagnosis	Clinical	62	43
	Electrophoresis of Hb	44	31
	Emmel test	37	26
Circumstances of the first diagnosis	Vaso-occlusive crisis	94	66
	Anemia	25	18
	Screening at the time of inclusion	14	10
	Fever	8	6
	Jaundice	2	1
	Neonatal screening	0	0
Confirmation of diagnosis by isoelectrofocusing	Yes	143	100
Chronic complications	Yes	31	22
Type of complications	Hip arthritis	13	11
	Stroke	7	5
	Right eye blindness	1	1
	Osteomyelitis	4	3
	Leg ulcer	3	2
	Other	3	2
Reasons for hospitalizations	Vaso-occlusive crisis	45	31
	Infectious episodes	34	24
	Anemia/blood transfusion	24	17
	Other causes	40	28
Presence of hepatomegaly	Yes	86	60
Presence of jaundice	Yes	126	88
Presence of splenomegaly	Yes	98	69
Spleen measurement (according to Hackett’s grade)	H0	45	31
	H1	16	11
	H2	34	24
	H3	24	17
	H4	15	10
	H5	9	6

*IQR* interquartile range, *Hackett’s grade* H0, non-palpable spleen, even in deep inspiration, *H1* Spleen palpable only on deep inspiration, *H2* Spleen palpable on mid-clavicular line, halfway between umbilicus and costal margin, *H3* The spleen expands towards the umbilicus, *h4* spleen descending below the navel, exceeding the line passing between the umbilicus and the pubic symphysis, *H5* spleen extending lower than class H4

### Comparison of data before and after the implementation of standardized and regular follow-ups

After 1 year of implementation of standardized and regular follow-ups, an overall reduction in the annual average of clinical complications has been observed, i.e., VOC, infectious episodes, acute chest syndrome, blood transfusions, and hospitalizations (Table 4). To evaluate the evolution of these parameters, the results were expressed as the percentage of the lower limit of the reference range due to the variation in reference ranges with age for the considered biological parameters. A significant increase in hemoglobin level and platelet count has been observed as well as a decrease in lymphocyte count. We did not observe statistical differences for the other hematological parameters (Table 5). Anemia was observed in 100% of our cohort and was severe and normocytic for 115 patients (80%).

**Table 4:** Comparison of acute complications of SCD before and after the Implementation of Standardized and Regular Follow-Ups

	Year 1	Year 2	*p*-value
Clinical Parameters	Follow-up without any intervention ***n*** = 143	Standardized and regular follow-up ***n*** = 143	
	Annual average [IQR]	Annual average [IQR]	
Vaso-Occlusive Crisis	3.9 [1–6]	1.1 [0–2]	< 0.001 (***)
Infectious episode	4.0 [2–6]	1.1 [0–1]	< 0.001 (***)
Hospitalization	3.8 [2–5]	1.2 [0–2]	< 0.001 (***)
Acute Chest Syndrome	1.0 [0–1]	0.0 [0–0]	< 0.001 (***)
Blood Transfusion	1.9 [1–3]	0.0 [0–1]	< 0.001 (***)

Significant *p*-values (≤ 0.05) appear in bold

*IQR* interquartile range (25–75%)

**Table 5:** Comparison of biological parameters for sickle cell patients before and after the Implementation of Standardized and Regular Follow-Ups

	Year 1	Year 2	*p*-value
Biological Parameters	Follow-up without any intervention Median (+/− SD)	Standardized and regular follow-up Median (+/− SD)	
HB (g/L)	50 (54 ± 20)	76 (76 ± 14)	**< 0.001**
RBC (× 10^6^/mm^3^)	2.0 (64 ± 25)	2.9 (86 ± 19)	**< 0.001**
HCT (%)	15 (61 ± 21)	23 (84 ± 15)	**< 0.00**
MCV (fL)	81 (129 ± 20)	82 (125 ± 13)	0.176
MCH (pg)	25 (20 ± 38)	25 (16 ± 35)	0.19
WBC (× 10^3^/mm^3^)	9.6 (374 ± 201)	8.4 (270 ± 109)	**< 0.001**
Lymphocytes (× 10^3^/mm^3^)	4.1 (364 ± 293)	3.2 (239 ± 145)	**< 0.001**
Neutrophils (× 10^3^/mm^3^)	4.7 (375 ± 205)	4.4 (369 ± 137)	0.554
Platelets (× 10^3^/mm^3^)	260 (248 ± 131)	328 (317 ± 108)	**< 0.001**

The averages of biological parameters represent the percentage of the normal lower limit of the reference range (% LLN) ± SD of the evolution of each patient in relation to themself

*HB* hemoglobin, *RBC* red blood cells, *HCT* hematocrit, *MCV* mean corpuscular volume, *WBC* white blood cells, *MCH* mean corpuscular hemoglobin

The therapeutic characteristics before the implementation of a regular follow-up are described in Table 6. None of the SCD children had been treated with hydroxyurea in the past. Applying the criteria for consensual indications of hydroxyurea treatment (≥2 acute chest syndromes per year, vaso-occlusive crisis ≥3 per year, severe anemia < 70 g/L**)** in the management of SCD, 94% of SCD children in the studied cohort required the introduction of hydroxyurea treatment.

**Table 6:** Therapeutic characteristics before regular follow-up applying sickle cell management recommendations (*n* = 143)

Variable	n	%
Prophylaxis		
Folic acid and oral penicillin	21	15
Routine vaccine	81	57
Pneumococcal vaccination (23 valent)	0	0
Antimalarial	0	0
Dewormers	0	0
Hydroxyurea treatment	0	0
Traditional treatment	96	67
Indicated for hydroxyurea treatment	134	94

**Fig. 4:**
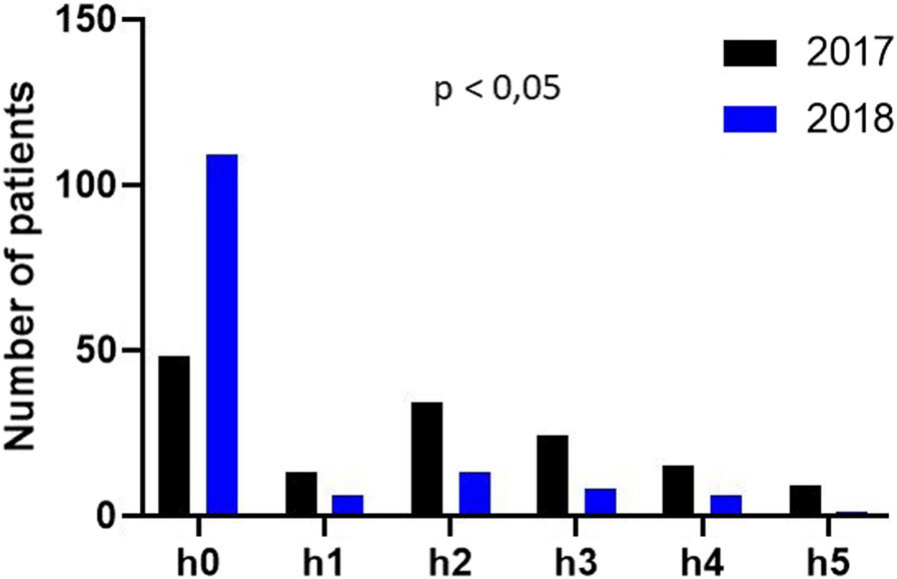
Comparison of splenomegaly before (2017) and after (2018) the implementation of a standardized and regular follow-up. Classification of splenomegaly according to Hackett’s grade: h0, non-palpable spleen, to h5, spleen extending lower than class h4

Comparison of the evolution of splenomegaly 1 year before and 1 year after the implementation of standardized and regular follow-ups showed a statistically significant difference (*p*-value < 0.001) (Fig. 4).

## Discussion

This study took place at the pediatric clinic of Mbujimayi where out-patient SCD care was standardized and assessed. A cohort of 143 SCD children with a median age of 10 years was followed for 2 years. In the first year, follow-up was limited to the management of acute complications without other specific interventions, and the second year included the implementation of standardized and regular follow-ups without the introduction of hydroxyurea. The results of this second year showed an overall reduction in acute complications and improvement of anemia. The establishment of a newly sickle cell reference center with evidence-based guidelines had a positive impact on reducing the morbimortality of SCD children even before the introduction of hydroxyurea and in the context of a remote city in the DRC.

In the context of poverty, the goal of equal access to healthcare can only be achieved if health policies guarantee effective care for all patients. As described in Mali, providing centers and units of competence is vital [36]. In the DRC, the few existing reference centers for SCD are not in remote areas but well in Kinshasa (Monkole Hospital and the SS Centre for Mixed Medicine and Anaemia (CMMASS), and Lubumbashi (SCD reference center at Nsendwe Hospital). In other African countries, for example, reference centers have also been mainly reported in capital-cities like Bamako, Mali (Centre for Research and control for SCD), Brazzaville, Republic of Congo (National Reference Centre for SCD) or Cotonou, Benin (National Sickle Cell Disease Centre) [37–39]. All these centers are almost entirely privately funded but have received government support in their implementation. In fact, until now, the management of SCD in sub-Saharan-Africa is not sustainable without external funding as it is the case with communicable diseases such as HIV/AIDS, malaria, and tuberculosis.

This study included SCD children 10-year-old on average for whom the initial diagnosis was not reported at birth. This initial diagnosis was mainly made based on clinical features, especially the presence of VOC or anemia at the age of 2 years on average. These results are consistent with those of other African authors who reported an average age of around 10 years in SCD patients and a first diagnosis at the age of 2 years or later with VOC reported as the most frequent mode of first diagnosis [40–45]. A major barrier was the absence of large-scale early-life screening and the high cost of screening with conventional methods i.e. isoelectric-focusing [14]. To strengthen and improve the diagnosis of SCD in health centers located in remote areas of sub-Saharan Africa, it would be desirable to use a point of care test. This has already been successfully deployed in comparable environments and could potentially enable early screening of SCD [44, 45].

Patients were subjected to a 2-year follow-up process including a monthly planned medical visit with a clinical and hematological assessment. The results of the first year of follow-up showed that sickle children without any specific medical intervention presented severe clinical and hematological pictures with an annual average of four VOCs, four infectious episodes, one acute thoracic syndrome, and four periods of hospitalizations. Annual averages of acute events and degradation of biological parameters similar to the results of this study, and in similar conditions of follow-up, have been reported in the literature [17, 40, 41, 46, 47].

The application of standardized and regular follow-up for 12 months showed encouraging results, with a significant reduction in acute events of the disease, i.e., a reduction in episodes of VOC, infectious episodes, blood transfusions, acute thoracic syndromes, hospitalizations, and an increase in the hemoglobin level from 50 to 70 g/L. The steady-state hemoglobin level of 50 g/L before the regular follow-up was lower than that previously reported for large cities of the DRC or other African countries (+/− 70 g/L) [40, 41, 48, 49]. This could be explained by the lack of optimal management of the disease in the past but, also, by other factors that should be explored. The prevalence of 47% underweight for height is disturbing. It suggests chronic malnutrition or severe chronic anemia, or both. Growth retardation remains common in children with SCD of SS / Sβ ° phenotypes in sub-Saharan Africa; it has been associated with anemia and hyperhemolysis [43].

Similar results were observed during the implementation of an SCD management program in a tertiary hospital in a remote area of Western India by a non-governmental organization. They registered 404 SCD patients between December 2015 and June 2017 and compared the uptake of proven interventions and indicators of disease severity from 1 year prior to registration until the end of the study (June 2018). After the introduction of standardized and regular monitoring, they observed a statistically significant decrease in VOC (277 vs. 53.4), hospitalizations number (49.8 vs. 42.2), and blood transfusions (27.4 vs. 17.8) [50].

Prospective cohort studies of SCD patients are rare in Africa due to barriers to medical monitoring [36]. However, a cohort study conducted in Benin in 2015 showed that the frequency of VOC was reduced to about one every 2 years, and some of the patients were crisis-free for as long as 5 years after implementing comprehensive healthcare management [51].

None of the enrolled children in this study were treated with hydroxyurea in the past while 94% of them had an indication for this therapy [13]. A Nigerian study reported also that 65% of 206 SCD patients had an indication for hydroxyurea treatment [52]. The efficacy and therapeutic benefits of hydroxyurea in SCD have been widely documented; it remains the appropriate basic treatment for its management and this study demonstrated that its side effects can be monitored [8, 13, 53–57]. However, in low- and middle-income countries, there are many barriers to hydroxyurea treatment. These barriers include ignorance, the non-prescription of the drug by doctors, and the cost of the drug [58, 59]. Hydroxyurea is rarely available in pharmacies in the DRC and this is noticeable in a remote area. The price of hydroxyurea in the DRC ranges from $10 to $35, with an average price of $15 for a 25-capsule 500 mg box. The annual cost of hydroxyurea treatment for a 25 kg SCD patient at the minimum dose of 20 mg/kg/day can be estimated at $215. Considering the purchasing power of most sickle cell patients, this price is prohibitive [60]. To overcome this problem of availability and accessibility to hydroxyurea, the policy options recommended by WHO should be implemented by the governments of African states, these include the regulation of price increase, the tax exemption for pharmaceuticals, the use of the evaluation of health technologies, and promotion of generic drugs [61]. The creation of reference centers and the organization of optimal management of SCD by doctors trained in SCD would certainly increase the knowledge, prescription, and awareness of hydroxyurea use. In an Indian study, the number of SCD patients on hydroxyurea increased from 4 to 98% after the implementation of a comprehensive program of SCD management in a remote tribal area [50].

If the creation of a reference center represented an awareness and an ongoing response to the management of SCD for the families concerned, however, the large number of patients lost during follow-up is worrying. While in Malawi, a prospective observational study in pediatric patients with SCD showed a loss of follow-up rate at 2 years under hydroxyurea treatment of 12.3% (23/187) [62], in our study, 40% of patients were lost to follow-up. This finding should prompt a rethinking of patient monitoring strategies and how to raise awareness of the disease.

It is widely accepted that SCD is associated with a very high mortality rate and particularly in Africa before the age of five [2] while among our studied population only 4 deaths (1.6%) were recorded. Globally, the mortality rate among children aged 5–14 years is 7.5 per 1000, among them, 98% (98–99) of all deaths in this age group occurred in low-income and middle-income countries, and in particular, in DRC this mortality rate is 104 per 1000 [63, 64]. The low mortality rate observed in our study could be attributed to the positive impact of medical follow-up or be underestimated among the population of the lost to follow-up. The effectiveness of care can be measured by the survival rate and this indicator should be part of the monitoring of the implementation of policies and care programs in Africa [2]. In Jamaica, the establishment of early diagnosis and simple prophylactic measures, i.e., oral penicillin and diagnosis of splenic sequestration, led to a significant reduction in SCD-associated deaths [16].

This study was based on free care to sickle cell patients to facilitate access to care and the implementation of medical follow-up including the use of simple and accessible means (the administration of folic acid and antibiotic prophylaxis). But this model has not been integrated into the primary healthcare system, such as is the case with the treatment of malaria, tuberculosis, or HIV that are properly managed at health center levels even in remote areas of sub-Saharan Africa. In a local study, 94% of parents of affected children had accepted annual medical insurance if the amount owed was less than $ 100 [17]. For the sustainability of the actions supported, an annual sickle cell health insurance program will be set up. However, this sustainability will also require the involvement of political authorities in favor of health care coverage and the integration of SCD in those priority areas, as is the case for HIV, tuberculosis, and malaria.

This study has certain limitations. The size and the level of severity of the disease observed in our cohort could be linked to selection bias. Another aspect already mentioned is the high proportion of patients lost to follow-up. Moreover, even if the treatment is free of charge when the clinical expression is moderate, it is very likely that families do not go to a medical center. A longer-term study would undoubtedly make it possible to approach these patients and overcome those biases.

## Conclusions

The creation of a sickle cell reference center for the regular monitoring of affected children and the application of management recommendations for this disease are possible and applicable in the context of a remote city in a country with low and middle income. Regular and rigorous monitoring including the application of simple and accessible measures based on international recommendations can help reduce the morbidity and mortality of these patients. The future challenges for improving patient care are however numerous. They relate, among other aspects, to the introduction of a simple and feasible means of early diagnosis and treatment with hydroxyurea. The implementation of this comprehensive care plan and in particular its sustainability requires strong political support. A final perspective is to advocate, at the government level, for free care for all sickle cell children to guarantee regular follow-ups.

## Data Availability

All data generated and/or analyzed during this study is available in a database from the corresponding author on reasonable request.

## Abbreviations

BCG: Bacillus Calmette–Guérin
CMMASS: SS Centre for mixed medicine and anemia
CNRD: National reference center for SCD
CRLD: Center for research and control for SCD
DRC: Democratic Republic of Congo
DPT: Diphtheria, Pertussis (whooping cough), and Tetanus
FU: Follow-up
Hb: Hemoglobin.
HCT: Hematocrit.
HIV: Human immunodeficiency viruses
IQR: Interquartile range
IEF: Isoelectrofocusing
LLN: Normal lower limit
MCV: Mean corpuscular volume.
MIBA: Bakwanga mining (Minière de bakwanga)
OPV: Oral polio vaccine
PNLCD: National SCD control program (Programme National de Lutte Contre la Drépanocytose)
RBC: Red blood cells
SCD: SCD
SD: Standard deviation
SMS: Short message service
UN General Assembly: United Nations General Assembly
USA: United State of America
VOC: Vaso-occlusive crisis
WBC: White blood
WHO: World health organization
